# Effect of vitamin D supplementation on metabolic syndrome parameters in adults: a systematic review of randomized controlled trials exploring the role of baseline vitamin D status and supplementation dose

**DOI:** 10.3389/fnut.2026.1974621

**Published:** 2026-10-27

**Authors:** Hind Alzarooni, Falak Zeb, Dimitrios Papandreou

**Affiliations:** 1 Department of Clinical Nutrition and Dietetics, College of Health Sciences, University of Sharjah, Sharjah, United Arab Emirates; 2 College of Sport Science, University of Kalba, Kalba, Sharjah, United Arab Emirates; 3 Research Institute of Medical and Health Sciences, University of Sharjah, Sharjah, United Arab Emirates

**Keywords:** insulin resistance, metabolic syndrome, randomized controlled trials, systematic review, vitamin D supplementation

## Abstract

**Introduction:**

Metabolic syndrome (MetS) is a cluster of cardiometabolic abnormalities including central obesity, dysglycemia, hypertension, and dyslipidemia. Vitamin D deficiency is highly prevalent among adults with MetS and may contribute to insulin resistance, inflammation, and adverse lipid profiles. Although vitamin D supplementation has been reported to improve MetS parameters, limited information is available on whether these effects differ by baseline vitamin D status or supplementation dose, information essential for clinical decision-making.

**Methods:**

This systematic review evaluated the effect of oral vitamin D supplementation on MetS components in adults and examined whether treatment effects appeared differ according to baseline vitamin D status and supplementation dose. A comprehensive search of PubMed/MEDLINE, Embase, Web of Science, and CENTRAL was conducted following PRISMA guidelines. Given the anticipated clinical and methodological heterogeneity across eligible trials, a narrative synthesis rather than meta-analysis was planned a priori. Risk of bias was assessed using the Cochrane Collaboration Risk of Bias Tool. Eligible RCTs included those evaluating oral vitamin D supplementation for at least 8 weeks in adults diagnosed with metabolic syndrome or presenting at least 3 of 5 MetS components. In addition, comparator groups included placebo or dietary-instruction-only controls. Outcomes of interest included waist circumference, triglycerides, HDL-cholesterol, blood pressure, fasting glucose, homeostatic model assessment of insulin resistance (HOMA-IR), glycated hemoglobin (HbA1c), and body mass index (BMI).

**Results:**

Eight randomized controlled trials (634 participants) met the full eligibility criteria and were included in the narrative synthesis. Vitamin D supplementation consistently increased serum 25(OH)D concentrations across trials. Significant improvements were reported in triglycerides and insulin resistance indices in a subset of trials, and in selected inflammatory markers, waist circumference, and BMI in individual studies, while blood pressure, HDL-cholesterol, and fasting glucose showed no consistent change across the majority of trials. Findings were most favorable among participants with baseline vitamin D deficiency and in trials using higher supplementation doses, although these patterns are exploratory, as no trial was powered for formal subgroup comparison. Risk-of-bias assessment indicated that seven of the eight evaluated trials had some concerns or high risk of bias in at least one domain, limiting the overall certainty of the evidence.

**Conclusions:**

Vitamin D supplementation shows slight benefit for specific MetS components, particularly among vitamin D-deficient adults, but effects were inconsistent across trials and metabolic parameters. Substantial variability in study design, dosing strategies, and population characteristics limits definitive conclusions. Adequately powered, standardized randomized trials are needed to clarify vitamin D's role in metabolic syndrome management.

**Systematic review registration:**

CRD420261421121.

## Introduction

MetS is a cluster of five cardiometabolic components namely central obesity, elevated triglycerides, reduced HDL-cholesterol, hypertension, and elevated fasting glucose which collectively elevate cardiovascular and metabolic disease risk. Diagnostic criteria have evolved over time, with the now widely used harmonized definition requiring any three of five components (1–3). Its burden has grown sharply worldwide: a recent modeling analysis spanning 198 countries estimated that global MetS prevalence roughly doubled between 2000 and 2023, reaching an estimated 1.54 billion affected adults by 2023, and rising further with age, urbanization, and income level (4). Mechanistically, insulin resistance and dysfunctional visceral adipose tissue sit at the center of MetS pathogenesis: expanding visceral fat behaves as an active endocrine organ, secreting pro-inflammatory cytokines and adipokines that impair insulin signaling systemically, while oxidative stress and chronic low-grade inflammation further compound metabolic dysregulation (4–7).

Vitamin D is a fat-soluble secosteroid, synthesized endogenously in skin on ultraviolet-B exposure or obtained from limited dietary sources, that undergoes sequential hydroxylation, first hepatic conversion to 25-hydroxyvitamin D 25(OH)D, the main circulating and storage form and then renal conversion to the biologically active hormone 1,25-dihydroxyvitamin D (calcitriol). Vitamin D receptor (VDR) expression extends beyond classical calcium-regulating tissues to pancreatic β-cells, skeletal muscle, and adipose tissue (8, 9). Several biological pathways have been proposed to link vitamin D to MetS components: vitamin D signaling has been shown to support pancreatic β-cell insulin secretion, promote glucose uptake in skeletal muscle, and modulate adipogenesis, lipid metabolism, and inflammatory cytokine output in adipose tissue (9–11). Vitamin D also negatively regulates the renin-angiotensin-aldosterone system, with deficiency linked experimentally to renin overexpression and consequent hypertension (12).

Despite this biological plausibility, the epidemiological and interventional picture is far less settled. Vitamin D deficiency itself is common worldwide, and appears disproportionately so among people with MetS: a global pooled analysis of close to 8 million participants estimated that around three-quarters of the world's population falls below the 30 ng/mL threshold often used to define insufficiency, with marked regional variation (13). Multiple observational studies have found vitamin D deficiency significantly more common among adults with MetS, with a graded inverse association between serum vitamin D concentration and MetS risk (14, 15). A recent meta-analysis of observational studies specifically in adults with MetS found that vitamin D deficiency was associated with significantly higher odds of MetS (OR 1.84, 95% CI 1.15–2.94) (16). Interventional evidence, however, has not consistently borne out these observational signals. A 2022 systematic review and meta-analysis of supplementation trials in adults with MetS described the evidence base as attracting extensive research interest but yielding inconsistent results across trials (17), and a meta-analysis focused specifically on lipid outcomes found no consistent benefit on total, LDL, or HDL cholesterol, with triglycerides in some analyses paradoxically rising rather than falling after supplementation (18). Individual trials illustrate this inconsistency directly; a double-blind RCT using weekly 50,000 IU vitamin D3 in adults with MetS reported significant reductions in triglycerides and improved insulin sensitivity measures (19), whereas a separate pilot RCT in a similar population found no effect of supplementation on any cardiovascular risk factor examined (20), and a trial restricted to postmenopausal women found supplementation improved the overall MetS risk profile (21). Three broadly comparable designs, three different conclusions. Reviewers attribute much of this inconsistency to systematic heterogeneity across trials. Differences in supplementation dose, baseline vitamin D status, treatment duration, and population characteristics all modify outcomes. An additional, less frequently considered source of heterogeneity is endogenous vitamin D synthesis. Because cutaneous production depends on ultraviolet-B exposure, serum 25(OH)D varies with latitude, season, and individual sun-exposure behavior. In supplementation trials, 25(OH)D may therefore change in placebo as well as intervention arms. Pooled effects have been shown to differ meaningfully between trials using ≤ 4000 IU/day and higher doses (17), and subgroup analyses in prediabetic and diabetic populations have reported larger glycemic benefits among participants who were vitamin D-deficient at baseline (22, 23). A 2025 systematic review of obesity-associated MetS identified population type, baseline status, dose, and duration as principal sources of heterogeneity (24).

This pattern-biologically plausible mechanisms and reflected in well-documented observational association, is complicated by interventional literature that remains equivocal and sensitive to both baseline vitamin D status and dose, pointing to a clear gap. Existing systematic reviews and meta-analyses in this space have generally been narrow in scope: some restrict analysis to a single outcome domain such as lipids (18) or endothelial function (25), others focus on obesity or diabetes-specific subpopulations rather than MetS as a whole (24, 26), and even the most recent reviews often pool a small number of trials without formally stratifying results by both baseline vitamin D status and supplementation dose simultaneously (25, 26). A 2022 systematic review and meta-analysis by Qi et al. (17) pooled an earlier and smaller set of trials in adults with metabolic syndrome using formal meta-analysis. The present review instead uses narrative synthesis, given substantial clinical and methodological heterogeneity across trials, and draws on a more recent body of evidence. Similarly, although Li et al. and Mirhosseini et al. (22, 23) showed that baseline vitamin D status and supplementation dose specifically affect glycemic outcomes, these findings were restricted to populations with type 2 diabetes or prediabetes rather than metabolic syndrome. The current review extends this dual-stratification approach to a broader set of outcomes relevant to metabolic syndrome as a whole, including lipid profile, glycemic control, anthropometric measures, and blood pressure, rather than a single outcome in a different population. This systematic review therefore aims to synthesize the available randomized controlled trial evidence on the effect of vitamin D supplementation, compared with placebo, on cardiometabolic outcomes in adults with MetS, and to determine whether these effects are modified by baseline vitamin D status (< 20 ng/mL vs. ≥20 ng/mL) and supplementation dose (< 4000 IU/day vs. ≥4000 IU/day). Serum 25(OH)D thresholds throughout this review follow the Endocrine Society clinical practice guideline, which defines deficiency as < 20 ng/mL (< 50 nmol/L), insufficiency as 20–29 ng/mL (50–75 nmol/L), and sufficiency as ≥30 ng/mL (≥75 nmol/L) (27). The 20 ng/mL threshold for baseline vitamin D status therefore corresponds to this deficiency cutoff, while the 4000 IU/day dose threshold corresponds to the Tolerable Upper Intake Level for vitamin D in adults established by the Institute of Medicine (28), marking the boundary between standard and high-dose supplementation. Notably, a higher threshold of ≥30 ng/mL (75 nmol/L) has been proposed as necessary for extra-skeletal benefits specifically (29); the present review retains the 20 ng/mL deficiency threshold for baseline stratification, given its established clinical basis, while acknowledging that a higher threshold may be more relevant to detecting extra-skeletal effects.

## Methods

This systematic review was conducted and reported according to the PRISMA (Preferred Reporting Items for Systematic Reviews and Meta-Analyses) guidelines and registered in PROSPERO (International Prospective Register of Systematic), Center for Reviews and Dissemination under registration number CRD420261421121.

### Search strategy and selection criteria

A systematic search was conducted using databases PubMed/MEDLINE, Embase, Web of Science, and the Cochrane Central Register of Controlled Trials (CENTRAL). Studies published between January 2000 and June 2026 were eligible for inclusion, consistent with the review's inclusion criteria. Manual searching of reference lists and citation tracking were conducted to ensure complete identification of eligible studies. The final selection of included studies was finalized on 22 June 2026. The search strategy was applied consistently across all databases without additional filters, following the PICOS criteria (Table 1).

**Table 1:** PICOS criteria used for search strategy.

PICOS criteria	Descriptions and search terms used for each criteria
Population	Adults aged ≥18 years diagnosed with MetS according to ATP III, IDF, or equivalent criteria, or adults presenting ≥3 metabolic syndrome components.
Intervention	Oral vitamin D supplementation (vitamin D2, vitamin D3, or active vitamin D analogs) of any dose, frequency, or regimen, with minimum duration ≥8 weeks.
Comparisons	Placebo or no supplementation.
Outcomes	At least one primary MetS parameter (waist circumference, triglycerides, HDL cholesterol, blood pressure, fasting glucose) or secondary outcomes (HOMA-IR, HbA1c, BMI, MetS resolution).
Search terms	“vitamin D” OR “vitamin D supplementation” OR “cholecalciferol” OR “ergocalciferol” OR “calcitriol” AND “metabolic syndrome” OR “cardiometabolic” OR “insulin resistance” OR “metabolic components” AND “randomized controlled trial” OR “randomized” OR “randomized” OR “clinical trial”
Study design	Randomized controlled trials (parallel-group or crossover with ≥4-week washout).

Non-randomized or quasi-experimental studies, observational studies (cohort, case-control, cross-sectional), systematic reviews, meta-analyses, narrative reviews, animal or *in vitro* studies, conference abstracts and non-English publications were excluded. Search results were exported to Mendeley.

Two reviewers screened titles, abstracts, and full texts according to the eligibility criteria, and independently verified the list of included and excluded studies; any disagreements were resolved by a third reviewer.

### Data extraction

A total of 1,240 records were identified through database searching. After removing 310 duplicates, 930 records remained for title and abstract screening. Following full-text assessment of 150 articles, 10 randomized controlled trials met the eligibility criteria at full-text review. One of these, a single-arm pilot study without a placebo or dietary-instruction comparator (30), was subsequently excluded from the evidence synthesis for not fully meeting the pre-specified PICOS comparator criteria, leaving 9 RCTs initially included in the narrative synthesis. A further trial [El Bilbeisi et al., 2021; (31)] was subsequently excluded after full data extraction, as its measured outcomes (serum calcium and parathyroid hormone) did not correspond to any pre-specified primary or secondary MetS parameter, leaving 8 RCTs in the final narrative synthesis. A flow diagram of the literature search and study selection is presented in Figure 1. Data extraction was performed by two reviewers with discrepancies resolved by consensus from a third reviewer. For each randomized controlled trial, detailed information was extracted including reference, publication year, country, study design, sample size, duration of intervention, vitamin D dosage and form, frequency of supplementation, baseline vitamin D status, metabolic syndrome criteria used, participant characteristics (age, sex distribution, BMI), and all reported MetS outcomes. Extracted outcomes included waist circumference, triglycerides, HDL cholesterol, blood pressure, fasting glucose, HOMA-IR, HbA1c, BMI, inflammatory markers, and MetS resolution when available. These 8 RCTs included a total of 634 participants diagnosed with MetS or presenting at least three MetS components. All trials evaluated oral vitamin D supplementation compared with placebo or no supplementation, with intervention durations ranging from 8 to 36 weeks. Missing or unclear data were requested from original investigators when necessary.

**Figure 1:**
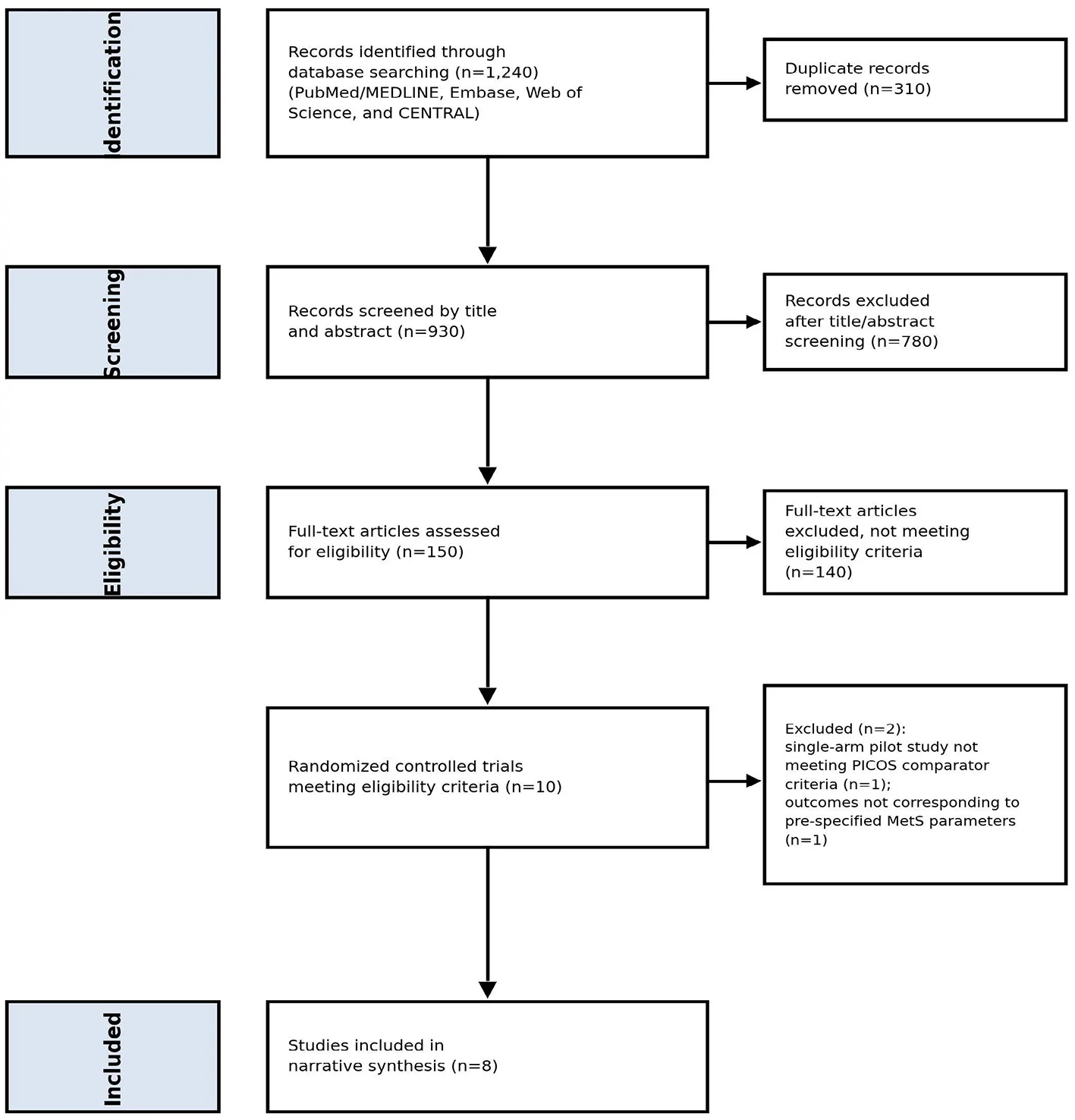
PRISMA flow diagram.

### Quality assessment

Assessment of reporting quality and risk of bias was also conducted by two reviewers according to the CONSORT (Consolidated Standards of Reporting Trials) guidelines, which include essential items related to the title, abstract, introduction, methodology, results, discussion, and other trial reporting domains. Risk of bias was assessed using the Cochrane Collaboration Risk of Bias Tool (RoB 1.0) across the seven domains including (a) random sequence generation, (b) allocation concealment, (c) blinding of participants and personnel, (d) blinding of outcome assessment, (e) incomplete outcome data, (f) selective reporting, and (g) other sources of bias.

Data were entered into Microsoft Excel for cleaning and preparation. Given the small number of eligible trials (*n* = 8) and substantial clinical and methodological heterogeneity in population characteristics, vitamin D formulation, dosing regimen, and outcome reporting, findings were synthesized narratively rather than statistically pooled. Study-level results for each MetS outcome were tabulated and compared descriptively across trials, consistent with recommended approaches for narrative synthesis when meta-analysis is not appropriate.

Sources of heterogeneity across trials, including baseline vitamin D status (< 20 ng/mL vs. ≥20 ng/mL) and supplementation dose (< 4000 IU/day vs. ≥4000 IU/day), were examined descriptively by comparing patterns of effect across studies grouped by these characteristics, rather than through formal statistical subgroup or dose-response meta-analysis, which was not feasible given the limited and heterogeneous set of included trials. Findings from studies at high risk of bias were interpreted with additional caution, consistent with a narrative sensitivity approach.

### Risk of bias assessment

This systematic review included eight randomized controlled trials that provided rigorous experimental data on the effects of vitamin D supplementation on MetS parameters. Quality assessment using the Cochrane Risk of Bias Tool revealed variability across studies: of the 8 included trials, one (21) was judged overall low risk of bias, four (19, 32–34) were judged to have some concerns, and three (20, 35, 36) were judged high risk. Ferreira 2020 demonstrated low risk of bias across all seven domains. Studies judged as “some concerns” were most often driven by unclear reporting of allocation concealment or incomplete outcome data (19, 32–34). Studies judged high risk were most frequently compromised by inadequate blinding of participants and personnel (20, 35, 36). Overall, most studies, seven out of eight, were judged to have “some concerns” or “high risk” in at least one domain. Figure 2 presents the detailed risk-of-bias assessment.

**Figure 2:**
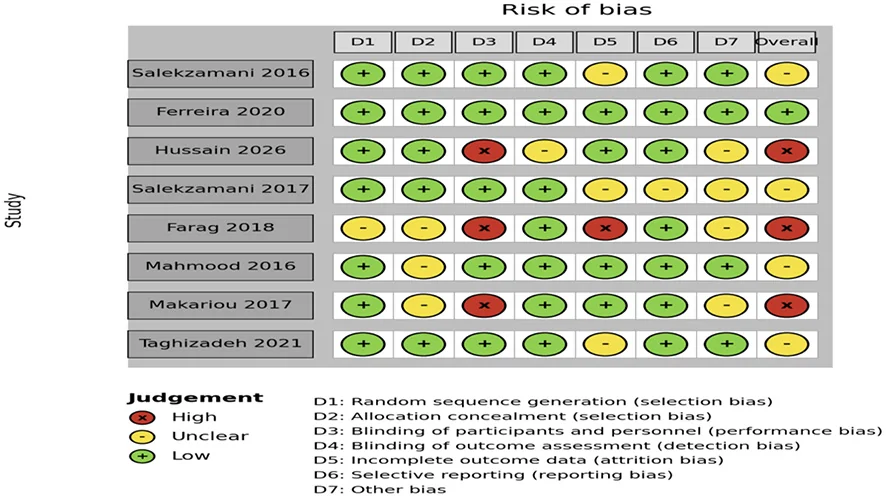
Risk of bias assessment.

## Results

### Effects of vitamin D supplementation on MetS parameters

Table 2 summarizes the characteristics of the included RCTs. In total, 8 RCTs were included, evaluating the effects of vitamin D supplementation (daily dosing regimens ranged from 1,000 to 2,000 IU/day; two trials used a weekly regimen instead (50,000–60,000 IU/week), and one trial administered two bolus doses of 200,000 IU at weeks 0 and 4) on MetS parameters in adults diagnosed with MetS or presenting ≥3 of the following five NCEP-ATP III/IDF diagnostic components: elevated waist circumference, elevated triglycerides, reduced HDL-cholesterol, elevated blood pressure, and elevated fasting glucose. All eight included trials administered vitamin D3 (cholecalciferol) rather than 25(OH)D directly. Four trials in which the original publication did not explicitly report D2 vs. D3. The trials were conducted across diverse geographic regions, including Brazil, Iran, Pakistan, Greece, India, and Iraq. The total number of participants was 634, with ages ranging from 30 to 65 years. Intervention durations varied from 8 to 36 weeks.

**Table 2:** Characteristics of the included studies assessing the effects of vitamin D supplementation on MetS parameters.

Reference	Country	Study design	Population	Age range (years)	Sample size	Vitamin D3 form and dose	Duration	Outcome type (Primary/Secondary)	Outcome measures	Key findings
Ferreira, 2020 (21)	Brazil	Double-blind, placebo-controlled RCT	Postmenopausal women with MetS	50–65	160 (80 VD, 80 placebo)	1,000 IU/day (D3)	36 weeks	Primary (stated study aim); power/sample-size calculation not reported	MetS components, insulin resistance	↑25(OH)D (+45.4% vs −18.5% placebo); ↓TG (−12.2%, *p* = 0.001); ↓Insulin (−13.7%, *p* = 0.008); ↓HOMA-IR (−17.9%, *p* = 0.007); lower risk of MetS (OR 0.42), hypertriglyceridemia (OR 0.43), hyperglycemia (OR 0.23)
Salekzamani, 2016(19)	Iran	Double-blind, placebo-controlled RCT	Adults with MetS	30–50	80 (40 VD, 40 placebo)	50,000 IU/week (D3)	16 weeks	Primary; *N* = 80 calculated for this outcome	MetS components, insulin resistance	↑25(OH)D (*p* < 0.001); ↓TG (between-group *p* < 0.001); → no change in FBS, insulin, HOMA-IR, Quicki, LDL-C, HDL-C, BP, anthropometrics
Salekzamani, 2017 (32)	Iran	Double-blind, placebo-controlled RCT [*post-hoc* analysis of (19)	Adults with MetS	30–50	80 (35 VD, 36 placebo analyzed)	50,000 IU/week (D3)	16 weeks	Secondary/*post-hoc* (same cohort as above; not independently powered)	Inflammation, adhesion molecules, CIMT	↓IL-6 (between-group *p* = 0.027); ↓VCAM-1 & ↓E-selectin (within-group only, *p* = 0.007/0.023; not significant between groups); → no change in hs-CRP or CIMT
Makariou, 2017(20)	Greece	PROBE (open-label, dietary-instructions control; no placebo)	Adults with MetS	39–63	50 (25 VD+diet, 25 diet-only)	2,000 IU/day (D3)	12 Weeks	Explicitly a pilot study — not powered for any outcome	Lipid profile, blood pressure, glycemic indices	→ no significant change in TG, HDL-C, LDL-C, FBG, HbA1c, HOMA-IR, or DBP in either group; SBP fell 3.7% in VD group (*p =* 0.05) vs 1.5% in control (NS), not different between groups
Farag, 2018(35)	Iraq	Parallel RCT (3 arms: VD, VD+PA, placebo)	Adults with MetS	30–50	90 randomized (70 analyzed: 24 VD, 21 VD+PA, 25 placebo)	2,000 IU/day (D3)	12 weeks	Primary; *N* = 180 (full 6-arm trial) planned, powered at 80% using fasting plasma glucose as the key variable	Lipid profile	VD+PA: ↓TC & ↓LDL-C (*p* = 0.002/0.017); VD alone: ↓TC, ↓HDL-C, ↓LDL-C, ↑TG, none significant; → no significant difference in TG/HDL-C across groups
Mahmood, 2016 (34)	India	Double-blind, placebo-controlled RCT	Adults with MetS	35–65	115 randomized (98 analyzed, 49 per group)	60,000 IU/week × 8 weeks → monthly × 4 months (D3)	24 weeks	Primary (stated aim: insulin resistance); power/sample-size calculation not reported	MetS components, insulin resistance	↓WC (*p* = 0.001) & ↓BMI (*p* = 0.001) in VD group; → no improvement in FBS, insulin, HOMA-IR, QUICKI; placebo group insulin rose (*p* = 0.03) and QUICKI fell (*p* = 0.02)
Taghizadeh, 2021 (33)	Iran	Quadruple-blind RCT (fortified yogurt vs placebo yogurt)	Adults with/without MetS consuming yogurt	30–50	139 total; 61 MetS subgroup (28 intervention, 33 control)	1,500 IU/day (nano-encapsulated, in yogurt), D3	10 weeks	Primary for full cohort (*n* = 139); table value is a MetS subgroup (*n* = 61), not independently powered	Inflammation, oxidative stress	In MetS subgroup: ↓hs-CRP (*p* = 0.036 between-group) & ↓PAB (*p* = 0.035 between-group); → no change in weight/BMI
Hussain, 2026 (36)	Pakistan	Randomized controlled trial	Adults with MetS	>19- No range was stated	78 (39 intervention, 39 control)	200,000 IU at week 0 & week 4 (D3)	8 weeks	Primary (registered main outcome); power/sample-size calculation not reported	Lipid profile	↑HDL-C (interventio*n* +0.09 vs control −0.02 mg/dL, *p* = 0.03), significant only in vitamin D-sufficient subgroup at baseline; → no significant change in TC, TG, LDL-C

RCT, Randomized Controlled Trial; MetS, metabolic syndrome; PROBE, prospective, randomized, open-label; FBS, Fasting Blood Glucose; HDL, High Density Lipoprotein; LDL, Low Density Lipoprotein; TG, Triglycerides; WC, Waist Circumference; hs-CRP, high sensitive C-Reactive Protein; BP, Blood Pressure.

Across the included studies, vitamin D supplementation consistently increased serum 25(OH)D concentrations. Improvements were observed in selected MetS components. Two studies (19, 21) demonstrated significant reductions in triglycerides. One study (21) showed significant reductions in fasting insulin and HOMA-IR. One study (34) showed significant reductions in waist circumference and BMI. Two studies reported improvements in inflammatory markers: (32) showed a significant reduction in IL-6, and (33) showed significant reductions in hs-CRP and pro-oxidant-antioxidant balance in the MetS subgroup. Two studies reported improvements in lipid profile: (35) showed significant reductions in total cholesterol and LDL-C in the vitamin D plus physical activity group, and (36) showed a significant increase in HDL-C. However, several of studies (19, 20, 34) showed no significant changes in blood pressure, HDL-cholesterol, or fasting glucose.

Notably, trials enrolling participants with confirmed baseline vitamin D deficiency (19, 21, 34) and trials using higher supplementation doses equivalent to roughly 7,000–8,500 IU/day (19, 32, 34) tended to show stronger improvements in metabolic parameters. In contrast, studies using lower doses ( ≤ 2,000 IU/day) or a short 8-week bolus regimen reported largely null results on insulin resistance and lipid profiles (20, 35, 36).

Classified explicitly by dose threshold, four trial-outcomes used a dose ≥4000 IU/day equivalent (19, 32, 34, 36), and all four reported a statistically significant improvement in at least one measured outcome. The remaining four trial-outcomes used doses < 4000 IU/day (20, 21, 33, 35); of these, three reported a significant improvement in a core MetS outcome (21, 33, 35), and one showed no significant change in any measured outcome (20). Baseline vitamin D status relative to the 20 ng/mL threshold was not consistently reported as a stratification variable across trials and could not be classified with the same confidence.

## Discussion

Vitamin D plays a central role in metabolic regulation, extending beyond its classical functions in calcium homeostasis and bone health (8). Emerging evidence suggests that vitamin D deficiency is associated with several metabolic abnormalities, including insulin resistance, dyslipidemia, and adverse cardiometabolic risk factors (14–16). In this systematic review, eight randomized controlled trials evaluating vitamin D supplementation in adults with MetS demonstrated variable improvements, with statistically significant improvement in at least one measured outcome reported in 7 of the 8 trials, in selected metabolic parameters. Across the included trials, vitamin D supplementation consistently increased serum 25(OH)D concentrations, confirming effective biological response to supplementation (19, 21). Improvements were most evident in triglycerides, fasting insulin, HOMA-IR, inflammatory biomarkers, and anthropometric measures such as waist circumference and BMI in a subset of trials (19, 21, 34). These findings align with mechanistic observations showing that vitamin D, acting through the vitamin D receptor (VDR), modulates insulin secretion and sensitivity, reduces inflammatory cytokine production, and influences adipocyte differentiation and lipid metabolism (7, 9, 10).

The mixed pattern of findings in this review is broadly consistent with earlier systematic reviews and meta-analyses in this field. A meta-analysis specifically in adults with metabolic syndrome found that vitamin D supplementation reduced insulin resistance and blood pressure but had no effect on lipid profile or HbA1c, concluding that overall evidence for benefit remains inconclusive (17), a pattern closely mirrored in this review. Similarly, meta-analyses focused specifically on lipid outcomes in MetS populations have found no significant effect of vitamin D supplementation on total, LDL-, or HDL-cholesterol, with one reporting a significant increase in triglycerides with supplementation (18), while another more recent meta-analysis found the opposite, stated that a significant triglyceride-lowering effect and HDL-cholesterol increase with daily dosing regimens (37), illustrating the genuine inconsistency in this literature even at the meta-analytic level. This discrepancy may partly reflect differences in how dosing regimen was handled analytically. This divergence is traceable to substantial heterogeneity from baseline imbalances among the trials pooled in the earlier meta-analysis, and to the more recent meta-analysis's decision to stratify trials by daily vs. non-daily dosing regimens. Differences in included trial sets over time, study population and reliance on formal statistical pooling, which is sensitive to which trials are included and how they are weighted, likely further contribute to the divergent conclusions across these reviews. None of the trials in the present review, nor evidently in the cited meta-analyses, were powered specifically for dose- or population-stratified comparisons, limiting the ability to adjudicate between these explanations directly. A GRADE-based systematic review similarly concluded that evidence does not support a benefit of vitamin D supplementation on triglycerides or waist circumference in MetS, citing low-to-very-low certainty in the available trials (38). Much of this apparent agreement reflects reliance on a largely overlapping, small pool of MetS-specific RCTs rather than independent confirmation. Consistent with this, none of the underlying primary trials were powered specifically for a triglyceride or waist-circumference endpoint, the same limitation noted in Table 2 of the present review. Broader meta-analyses of glycemic outcomes in related populations (prediabetes, type 2 diabetes) have shown similarly discrepant results: some report no significant effect of vitamin D on insulin sensitivity (39), while others report significant improvements in HbA1c, fasting glucose, and HOMA-IR, particularly in vitamin D-deficient or high-dose subgroups (22, 23). These broader reviews disagree with each other, and partly with the present review, because they define different populations (a broad “at risk” population vs. prediabetes/type 2 diabetes specifically) and use different outcome measures (the gold-standard hyperinsulinemic-euglycemic clamp vs. HOMA-IR); the reviews reporting benefit also deliberately excluded the bolus-dosing regimens used by two of the trials included in the present review. A meta-analysis in 14 studies that examined endothelial and adhesion markers, despite pooling substantially more participants (1,253) than the single relevant trial in the present review, the authors found improvement in von Willebrand factor but no significant effect on VCAM-1, E-selectin, or ICAM-1 (25). The researchers argued that these represent genuine null effects rather than an artifact of the smaller sample in this review, and consistent with the within-group-only improvements in adhesion molecules observed in another included trial (32) within this review. The present review therefore largely confirms, rather than contradicts, the pattern established in earlier syntheses: vitamin D supplementation reliably corrects biochemical deficiency but produces inconsistent, often null, effects across individual metabolic syndrome components, with meta-analytic evidence itself divided on direction of effect for lipid outcomes specifically. Where this review may extend prior work is in highlighting subgroup-level patterns, particularly around baseline vitamin D status and dose consistent with dose-response relationships described in prior epidemiological (40) and interventional (23) meta-analyses, which may help explain why pooled meta-analyses across heterogeneous populations often report weak or null average effects.

It is important to give proportionate weight to the null and inconsistent results in this review rather than emphasizing only the positive findings. Of the eight included trials, several reported no significant improvement in one or more core metabolic syndrome components. Blood pressure did not improve significantly in most trials that assessed it, HDL-cholesterol changed inconsistently across studies, and fasting glucose remained unchanged in the majority of trials (20, 34). Rather than treating these as isolated negative results, they should be read as the dominant pattern in the evidence base, one that mirrors the broader literature: several independent meta-analyses of vitamin D supplementation in MetS or related populations have reached similarly cautious or null conclusions regarding lipid and glycemic outcomes (17, 18, 26, 38).

Several plausible biological mechanisms link vitamin D to MetS components and may also help explain why the observed effects across trials were inconsistent (10). Vitamin D also appears to interact with the renin-angiotensin-aldosterone system and endothelial function, offering a mechanistic basis for the modest blood pressure effects observed in a minority of included trials (12). In adipose tissue specifically, vitamin D acts through both classical nuclear VDR pathways and non-classical pathways (including AMPK/SIRT1 signaling and microRNA regulation) to influence adipogenesis, lipid metabolism, and inflammatory responses (11, 41).

Whether the statistically significant biomarker changes observed in this review are clinically meaningful is uncertain. Triglyceride reductions reported in a subset of trials (19, 21) are of uncertain clinical significance given that broader meta-analyses in MetS populations disagree on both the direction and magnitude of vitamin D's effect on triglycerides (18, 37). This inconsistency was also evident within a single trial: one three-arm study found that vitamin D produced significant reductions in total cholesterol and LDL-cholesterol only when combined with structured physical activity, whereas vitamin D supplementation alone did not reach significance for any lipid parameter (35), suggesting that concurrent lifestyle intervention may influence whether vitamin D's lipid effects become detectable. The HDL-cholesterol increase reported in one included trial (36) was statistically significant but modest in absolute terms, and its significance was confined to participants who were vitamin D-replete at baseline rather than those who were deficient, further limiting its clinical applicability. Overall, the biomarker improvements identified in this review should be interpreted as signals warranting further investigation rather than as evidence of clinically actionable benefit, consistent with the low-to-very-low certainty of evidence reported in recent GRADE-based syntheses of this literature (38).

Substantial heterogeneity across the included trials likely contributes to the inconsistency of findings. Trials differed in vitamin D formulation (oral drops, pearls/capsules, and fortified yogurt using nano-encapsulation technology), dosing regimen (daily doses ranging from 1,000 to 2,000 IU, weekly doses of up to 50,000 IU, and bolus doses as high as 200,000 IU), and intervention duration (8 to 36 weeks).These differences in dosing frequency and total dose that a large network meta-analysis suggests can meaningfully affect the efficiency of raising circulating 25(OH)D concentrations, with roughly 2,000 IU/day (or equivalent monthly dosing) generally required to achieve optimal serum levels (42). Baseline vitamin D status also varied considerably across trials, consistent with global data showing wide regional and seasonal variation in vitamin D deficiency prevalence, with higher rates at higher latitudes and during winter months (13). Additional heterogeneity arose from differences in obesity severity across study populations and from geographic differences across the study sites (Brazil, Iran, Pakistan, Greece, India, and Iraq), both recognized as significant modifiers of vitamin D status and metabolic outcomes (13, 16).

Patterns suggesting that participants with baseline vitamin D deficiency or those receiving higher supplementation doses showed stronger metabolic improvements should be interpreted as exploratory observations rather than confirmed effects, as none of the included trials were specifically powered for subgroup comparison by baseline vitamin D status or dose. These patterns are nonetheless broadly consistent with dose-response relationships described in prior epidemiological meta-analyses (40) and with subgroup analyses from interventional meta-analyses showing stronger glycemic benefit in vitamin D-deficient individuals and with higher supplementation doses (22, 23). Age may also modify the vitamin D-MetS relationship, as one large cross-sectional study found a significant inverse association between vitamin D and MetS in older adults but not in middle-aged adults (15). However, age was not used as a formal stratification variable in the present review; none of the eight included trials reported age-stratified results, and splitting an already limited evidence base of eight trials by a third variable, in addition to baseline vitamin D status and dose, was judged unlikely to produce interpretable subgroups. Formal subgroup or dose-response meta-analysis within this review would be required to substantiate these patterns further.

Several factors may explain why fasting glucose and blood pressure failed to improve significantly across most trials. First, in several studies, baseline fasting glucose values were close to normal despite participants meeting metabolic syndrome criteria on other components, potentially limiting the ability to detect a glucose-lowering effect (32). Second, many trials failed to raise serum 25(OH)D to the range proposed as necessary for extraskeletal benefits, even where statistically significant increases from baseline were achieved; network meta-analytic data suggest that approximately 2,000 IU/day (or monthly equivalent dosing) is typically needed to reach optimal serum concentrations, a threshold not consistently met across included trials (42). Third, blood pressure regulation by vitamin D appears to depend substantially on the renin-angiotensin-aldosterone system and endothelial function, and effects have been most pronounced with higher doses over shorter durations in prior systematic reviews, which may explain why some of the included trials using lower or more prolonged dosing regimens showed weaker blood pressure effects (43).

## Strengths and limitations

This review has several strengths, including a comprehensive search of four major databases with manual reference and citation tracking, PROSPERO registration and PRISMA-adherent reporting, formal risk-of-bias assessment, and a focus on adults meeting harmonized metabolic syndrome criteria that allowed lipid, glycemic, anthropometric, and inflammatory outcomes to be considered together within a single evidence base. Descriptive exploration of patterns by baseline vitamin D status and supplementation dose, an angle often absent from narrower prior reviews, is a further strength. Several limitations should nonetheless be acknowledged. Substantial heterogeneity in study design, vitamin D formulation, dosing regimen, treatment duration, and population characteristics limited direct comparability across trials, and the small number of eligible RCTs (*n* = 8), most with modest sample sizes, precluded formal meta-analytic pooling; findings were therefore synthesized narratively, and reported effects should be interpreted cautiously rather than as precise estimates. Confounding factors such as diet, physical activity, and sun exposure were not consistently controlled across trials, potentially influencing observed effects (34). Changes in body weight were inconsistently reported and, to our knowledge, not adjusted for in the outcome analyses of the included trials. Finally, restriction to English-language publications and the modest number of included trials raise the possibility of language and publication bias. Overall, vitamin D supplementation may offer selective metabolic benefits, particularly for triglycerides, insulin resistance indices, and inflammatory markers, though effects on blood pressure, HDL-cholesterol, and fasting glucose remained inconsistent, underscoring the need for standardized, adequately powered trials exploring dose-response relationships.

## Future directions

Future research should prioritize adequately powered, multicenter randomized controlled trials that stratify participants a priori by baseline vitamin D status and supplementation dose, using standardized dosing protocols and sufficient follow-up duration to capture sustained metabolic effects. Trials should also report age-stratified outcomes and control more consistently for diet, physical activity, and sun exposure, allowing future meta-analyses to disentangle the sources of heterogeneity identified in the current evidence base. Such studies would help clarify whether vitamin D supplementation offers a genuine, clinically meaningful benefit for MetS management, particularly among vitamin D-deficient populations.

## Conclusion

Vitamin D supplementation showed suggestive but not conclusive benefit for selected components of MetS, particularly triglycerides, insulin resistance indices, and inflammatory biomarkers, with improvements most evident among vitamin D-deficient participants and those receiving higher or longer-duration supplementation; effects on blood pressure, HDL-cholesterol, and fasting glucose were inconsistent. Given the small number of available trials, substantial heterogeneity, and reliance on narrative rather than meta-analytic synthesis, these findings should be regarded as exploratory. Standardized dosing protocols, larger and adequately powered trials, and longer follow-up are needed before vitamin D supplementation can be recommended as a component of MetS management.

## Data Availability

The raw data supporting the conclusions of this article will be made available by the authors, without undue reservation.
